# Quality of life of veterinary residents in AVMA-Recognized Veterinary Specialty Organizations using the WHOQOL-BREF instrument

**DOI:** 10.1371/journal.pone.0268343

**Published:** 2022-05-12

**Authors:** Jennifer L. Jaworski, Lori A. Thompson, Hsin-Yi Weng

**Affiliations:** 1 Animal Allergy & Dermatology Center of Indiana, Indianapolis, Indiana, United States of America; 2 Department of Comparative Pathobiology, College of Veterinary Medicine, Purdue University, West Lafayette, Indiana, United States of America; PLOS (Public Library of Science), UNITED KINGDOM

## Abstract

**Objective:**

To assess whether there is an association between veterinary specialty and the quality of life of residents in AVMA-Recognized Veterinary Specialty Organizations^™^ using the WHOQOL-BREF instrument.

**Methods:**

This cross-sectional study used an online survey and data collection service for administration of the survey to veterinary residents during April 2021 to June 2021. Veterinary residents were contacted through their respective AVMA-Recognized Veterinary Specialty Organization^™^ and through social media. Overall quality of life along with the domains of Physical Health, Psychological Health, Social Relationships, and Environment were measured using the WHOQOL-BREF instrument. Additionally, data on the demographics of participants were collected and investigated as potential confounders. Mean standardized scores (0 to 100) were compared among the specialties using the general linear model.

**Results:**

792 residents from 21 veterinary specialties were included in the analysis. The results showed that overall quality of life and all four domains varied significantly among specialties after adjusting for significant demographic variables (all *P*s < 0.001). The mean standardized overall quality of life score was 54.3, ranging from 31.8 in Emergency and Critical Care to 56.3 in Laboratory Animal. The mean standardized quality of life scores were lowest for Psychological Health (50.3), followed by Social Relationships (55.0), Environment (61.4), and Physical Health (62.6). Residents in Emergency and Critical Care had the lowest adjusted average scores in all quality of life domains. Residents in Internal Medicine, Anesthesia and Analgesia, and Surgeons had lower quality of life scores across several domains when compared to other specialties.

**Clinical relevance:**

This study provides insight into the mental health and general well-being of veterinary residents. The results can assist veterinary specialty organizations, universities, and mentors in developing appropriate supporting programs for residents. The results can also assist residents in recognizing and more efficiently caring for their individual mental health and well-being.

## Introduction

In recent years, the mental health and overall well-being of veterinarians, starting from their time in veterinary school [1–3] and continuing throughout their professional careers [4, 5], has received increased attention. During veterinary school, students are subjected to a wide variety of academic [6] and non-academic stressors [7] and report high levels of anxiety throughout their duration of schooling [8]. In one study of 573 students currently enrolled at 16 different Veterinary Colleges in the United States, 30% reported having seriously thought about suicide and 5% reported having made a suicide attempt [3]. After completing veterinary school, veterinarians continue to be at higher risk of depression compared to other occupational groups [9]. A review of more than 11,000 veterinarian deaths over a 36 year period in the United States found that male veterinarians were 2.1 times and female veterinarians were 3.5 times as likely as the general population to die by suicide [4].

After veterinary school, veterinarians have the option to pursue board-certification in a veterinary specialty. This entails several years of further training in a specific area of veterinary medicine and the passing of an examination evaluating their understanding and skills in that specific specialty [10]. Currently, there are 22 American Veterinary Medical Association-Recognized Veterinary Specialty Organizations^™^ (AVMA-RVSOs) [10]. Approximately 15% of veterinarians have received their board certification [10, 11]. The mental health and well-being of veterinarians during their residency programs has not been extensively studied.

Quality of life (QOL) is defined by the World Health Organization Quality of Life (WHOQOL) Group as “an individual’s perceptions of their position in life in the context of the culture and value systems in which they live and in relation to their goals, expectations, standards, and concerns” [12]. The World Health Organization developed the life assessment instrument WHOQOL-BREF to measure QOL of an individual instead of only measuring the impacts of a disease by traditional health indicators such as mortality and morbidity [13–15]. This assessment instrument allows for a subjective evaluation focused on the respondent’s perceived QOL [12]. The WHOQOL-BREF instrument was selected for this study as it has been widely used in various populations [13, 15–17], including in previous studies looking at the overall QOL of students in medical school or medical residencies [18–21].

The purpose of this study was to use the US English version of the WHOQOL-BREF instrument on residents in AVMA-RVSOs to determine if there were differences in QOL among veterinary residents in different specialties after controlling for age, gender, marital status, and health status. To the best of the authors’ knowledge, this is the first study using the WHOQOL-BREF instrument on veterinary residents in AVMA-RVSOs. The results of this study will help institutions involved in veterinary residency programs understand the mental health challenges of their residents and develop appropriate supporting programs. Veterinary residents and veterinarians may find a self-assessment instrument helpful in navigating career choices.

## Materials and methods

This cross-sectional study was conducted among individuals self-identified as veterinary residents in an AVMA-RVSO during April 2021 to June 2021. This study was reviewed by Pearl IRB, LLC (an independent Institutional Review Board) and was determined to be exempt. The Checklist for Reporting of Results of Internet E-Surveys (CHERRIES) [22] was followed. For this study, AVMA-RVSOs were categorized into 13 groups (Table 1).

**Table 1:** Veterinary residency specialty groups and distribution in the study population (N = 792).

Specialty groups	N	%
American College of Veterinary Anesthesia and Analgesia	45	5.7
American College of Veterinary Behaviorists	20	2.5
American Veterinary Dental College	21	2.7
American College of Veterinary Dermatology	39	4.9
American College of Veterinary Emergency & Critical Care	108	13.6
American College of Veterinary Internal Medicine	106	13.4
American College of Laboratory Animal Medicine	87	11.0
American College of Veterinary Ophthalmologists	30	3.8
American College of Veterinary Pathologists	94	11.9
American College of Veterinary Radiology	92	11.6
American College of Veterinary Surgeons	76	9.6
American College of Zoological Medicine	24	3.0
Others^a^	50	6.3

^a^Including: American Board of Veterinary Practitioners (N = 16); American Board of Veterinary Toxicology (N = 3); American College of Poultry Veterinarians (N<3); American College of Theriogenologists (N = 9); American College of Veterinary Clinical Pharmacology (N = 4); American College of Veterinary Microbiologists (N<3); American College of Veterinary Nutrition (N = 4); American College of Veterinary Preventive Medicine (N = 6); American College of Veterinary Sports Medicine and Rehabilitation (N = 5); American College of Animal Welfare (N = 0).

Each AVMA-RVSO was approached by email requesting distribution of the WHOQOL-BREF instrument to active residents. Twelve AVMA-RVSOs emailed the survey to enrolled residents. Four AVMA-RVSOs (Dental, Ophthalmologists, Surgeons, and Zoological Medicine) with active residents in accredited residency programs could not share external communications with residents and declined to participate. Six AVMA-RVSOs (Animal Welfare, Microbiologists, Poultry, Sports Medicine and Rehabilitation, Theriogenologists, and Preventative Medicine) did not have active residents in fully accredited residency programs and did not participate. Individuals who self-identified as residents in these AVMA-RVSOs were included in this study. Residents were also contacted through social media and by email when contact information was available online. The study survey was constructed and distributed through SurveyMonkey. Informed consent was provided in the cover page and participants had to click agree before proceeding to the survey. The survey was anonymous, and the survey link was always the same therefore participants were not tracked on whether they responded to the survey through email or social media.

The WHOQOL-BREF instrument is a self-administered questionnaire, compromised of 26 items to assess the individual’s perception of overall QOL (two items) and four major QOL domains: Physical Health (seven items), Psychological Health (six items), Social Relationships (three items), and Environment (eight items). A five-point Likert scale, ranging from 1 (Not at all, Very poor, Very dissatisfied, Never) to 5 (Completely, Very good, Very satisfied, An extreme amount, Extremely, Very well), was used to score each item. The raw domain score was then converted into a standardized score, ranging from 0 to 100, with a higher score indicating a higher QOL, according to the published scoring instruction (Eq 1) [12, 14]. If no more than one item from Physical Health or Environment domains was missing, a standardized domain score was calculated by replacing missing values with a person-specific average. If two or more items were missing in these two domains, or if any items were missing in the Psychological, Social Relationships, or overall QOL domains, a domain score for that respondent was not calculated [12, 14].


$$\text{Standardized}\;\text{score}=[\frac{\left(Raw\;score-\;lower\;limit\;of\;the\;domain\;score\right)}{Range\;of\;the\;domain\;score}]\times 100$$ (1)

Before beginning the WHOQOL-BREF instrument, the participants were asked a screening question to exclude individuals not self-identified as current residents in an AVMA-RVSO. Additional information about the participants including specialty program in which they were currently enrolled, gender, age (grouped), marital status, and if they were currently ill, was also collected. Participation was voluntary and participants were not excluded if they did not answer all questions. For each analysis, participants were included if they responded to related questions.

Descriptive statistics, including mean ± standard deviation (SD) for standardized QOL scores and frequency (%) for specialty programs, age (grouped), gender, marital status, and current health status were reported. To protect privacy, absolute counts were not reported for any groups with less than three participants. Age (grouped), gender, marital status, and current health status were investigated for their associations with QOL measurements in the univariate analyses using ANOVA. Significant covariates identified in the univariate analyses were included in the general linear models for the comparisons of the mean standardized QOL scores among the specialty programs. Adjusted mean standardized domain scores and corresponding standard errors were reported. All the analyses were done separately for each of the four QOL domains and the overall perception of QOL. Statistical significance was set at *P* < 0.05. The presented p values were adjusted using the Bonferroni method.

## Results

**Fig 1:**
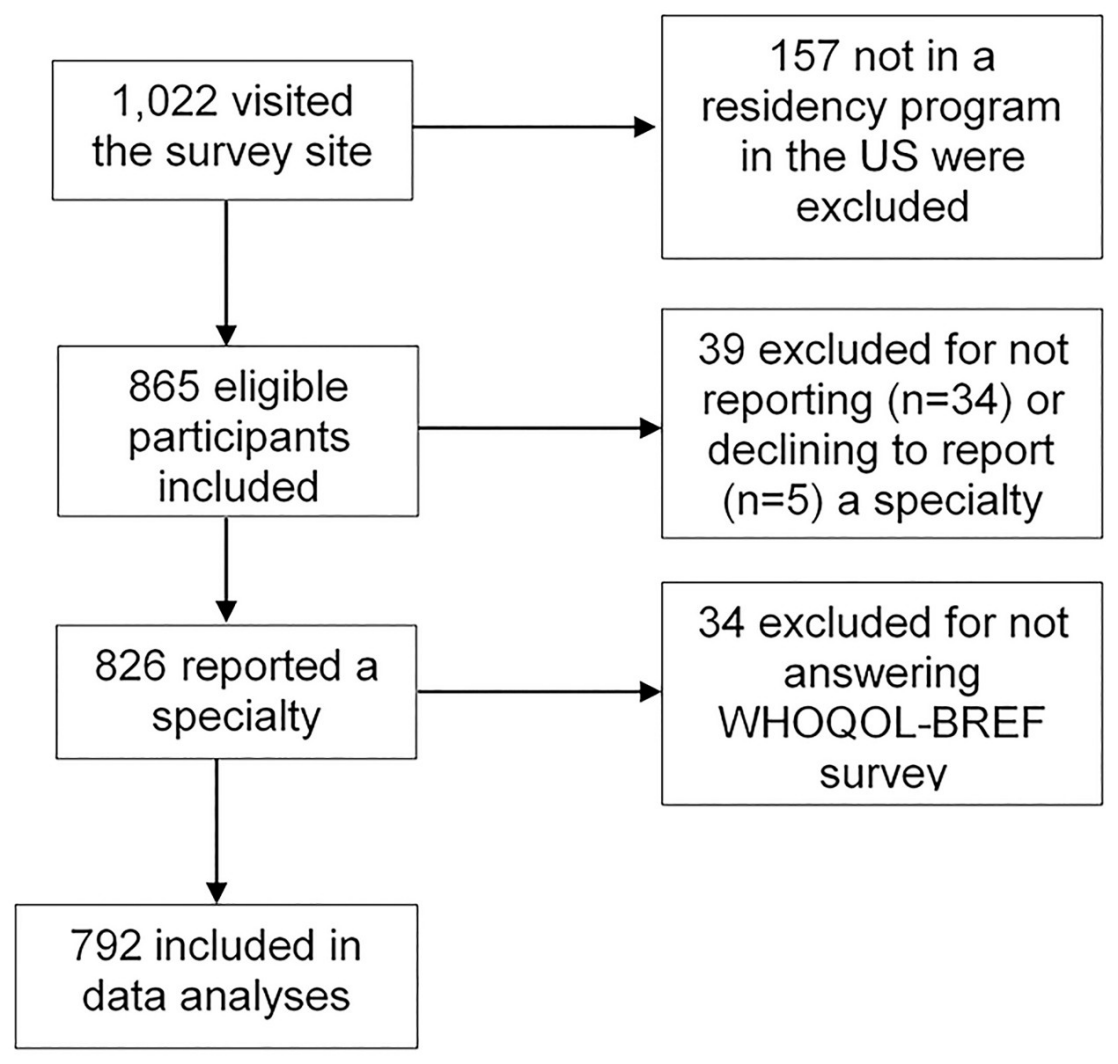
Flow-chart depicts the inclusions and exclusions of the study participants.

One thousand and twenty-two individuals visited the survey site, and among them 792 were included in the analyses (Fig 1). The breakdown and distribution of specialty groups in the study population are presented in Table 1. American College of Animal Welfare was not part of the study, as no residents from this specialty participated in the survey.

Demographic variables including gender, age (grouped), marital status and if the participant was ill at the time of the survey are presented in Table 2. The majority of participants were female (76.8%), 30 to 35 years old (52.3%), single (53.0%) and healthy (96.2%) at the time of the survey.

**Table 2:** Descriptive statistics of the demographic variables of the study participants.

Demographics	Frequency (%)
Age	
24–29	251 (31.7)
30–35	414 (52.3)
36–41	92 (11.6)
>41	34 (4.3)
Gender^a^	
Male	182 (23.2)
Female	604 (76.8)
Marital status	
Single	417 (53.0)
Married	289 (36.7)
Living as married	63 (8.0)
Other^b^	18 (2.3)
Currently ill	
Yes	30 (3.8)
No	752 (96.2)

^a^Non-binary (N<3)

^b^Including: Divorced (N = 16); Separated (N<3).

The results of the univariate analyses assessing the association between demographic variables and QOL measurements are presented in Table 3. Mean ± SD of the standardized QOL scores are reported. Marital status was significantly associated with QOL in all domains. Current health status was significantly associated with overall QOL (P < .001), Physical Health (P < .001), and Psychological Health (P = .011). Male residents had a higher score in Psychological Health than female residents (*P* = .009). Similarly, age of residents was only significantly associated with Psychological Health (*P* = .001). Significant covariates identified in the univariate analyses were included in the general linear models.

**Table 3:** Univariate analyses to assess the association between demographic variables and QOL measurements. Mean ± SD of standardized QOL scores are reported.

	Quality of Life domains				
	Overall perception	Physical health	Psychological health	Social relationships	Environment
Age	N = 791	N = 762	N = 751	N = 762	N = 762
24–29	53.5±22.7	62.3±15.8	48.7±18.6	53.8±22	60.9±15.6
30–35	55.5±23.3	63.3±16.2	51.4±18.3	56.4±21.9	61.8±15.8
36–41	50.4±23	59.5±16.5	45.1±19.7	51±20.1	60.5±14.2
>41	51.1±25.4	60.3±18.7	59.1±19.8	57.6±24.1	62.7±16.9
P-value^a^	.205	.187	.001	.119	.793
Gender	N = 786	N = 757	N = 747	N = 757	N = 757
Male	54.6±23.3	64.5±16.0	53.4±18.7	53.9±21.1	61.3±15.5
Female	53.8±23.2	61.8±16.3	49.2±18.7	55.2±22.2	61.4±15.6
P-value^b^	.701	.052	.009	.482	.952
Marital status	N = 787	N = 759	N = 749	N = 759	N = 759
Single	52.7±23.1	61.1±16	61.1±16	52.2±22.4	59.7±14.7
Married	56.4±23.2	64.8±16	64.8±16	58.2±20.5	64.3±16.1
Living as married	55.4±22.5	63±17.5	63±17.5	63.1±20.8	62.6±15.3
Other^c^	43.8±23.2	53.8±15.9	53.8±15.9	44±22.1	51.9±20.8
P-value^a^	.044	.004	.009	< .001	< .001
Currently ill	N = 782	N = 753	N = 743	N = 753	N = 753
Yes	37.9±25.5	46.1±15.5	38.1±24.9	49.4±29.6	58.6±18.3
No	54.9±22.8	63.2±15.8	50.7±18.3	55.2±21.5	61.5±15.5
P-value^b^	< .001	< .001	.011	.304	.329

^a^Derived from ANOVA analyses

^b^Derived from 2-sample t-tests

^c^Including Divorced and Separated

**Fig 2:**
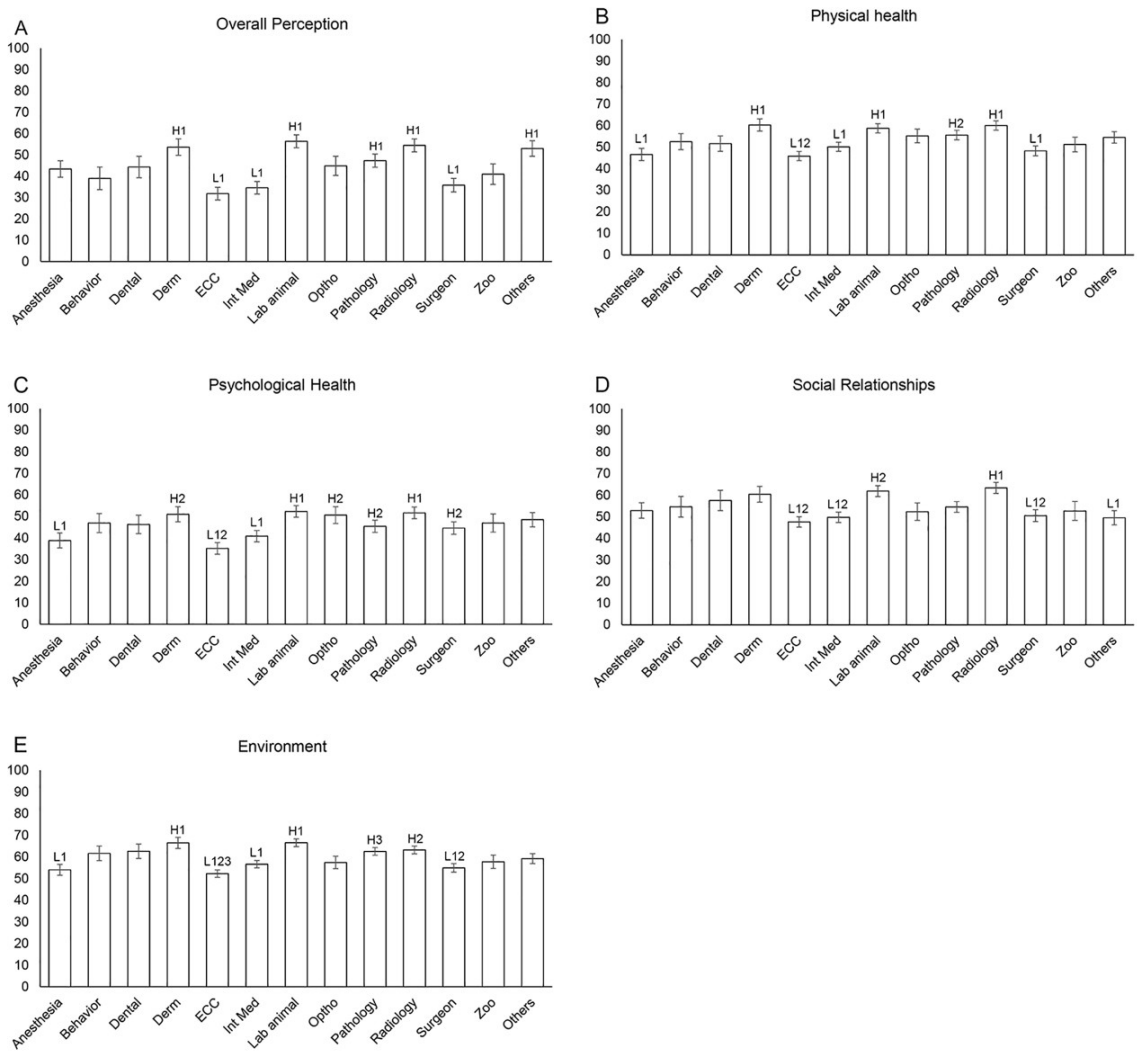
Bar charts comparing the mean (± SE) standardized QOL domain scores (A to E) among 13 veterinary resident specialty groups. H and L indicate significant comparison (i.e., Bonferroni adjusted *P*<0.05) between high QOL (H) and low QOL (L) scores. The numbers further group significant results together. For example, the mean Physical health score for ECC is significantly lower (L12) than the mean scores for Dermatology (Derm; H1), Lab animal (H1), Radiology (H1), and Pathology (H2). P-values and SE are derived from general linear models, adjusting for significant covariates identified in the univariate analyses.

The results of the 13 specialty groups in overall perception of QOL and each individual domain (Physical Health, Psychological Health, Social Relationships, and Environment) are shown in Fig 2. The means and standard errors (error bars) were estimated by the general linear models and were adjusted for significant covariates identified in the univariate analyses. Covariates included were marital and current health status for overall QOL and Physical Health domains; age, gender, marital status, and current health status for Psychological Health domain; marital status for Social Relationships and Environment domains. The mean (SD) standardized scores were 54.3 (23.1) for overall QOL, 62.6 (16.1) for Physical Health, 50.3 (18.8) for Psychological Health, 55.0 (21.9) for Social Relationships, and 61.4 (15.6) for Environment domains. There are significant comparisons (P < 0.05) between the five highest scoring specialties (Laboratory Animal (56.3), Radiology (54.4), Dermatology (53.6), Others (53.0), and Pathology (47.3)) and three lowest scoring specialties (Emergency and Critical Care (ECC) (31.8), Internal Medicine (34.5) and Surgeons (35.7)) for overall QOL.

The labels represent the significant comparisons (i.e., Bonferroni adjusted *P* <0.05), between low (L) and high (H) QOL scores. The letter and number together indicate the corresponding significant pairs. For example, L123 for ECC in the Environment domain indicates that ECC is significantly different from Dermatology (H1), Lab animal (H1), Radiology (H2), and Pathology (H3). Likewise, L1 for Anesthesia in the Environment domain indicates that Anesthesia is significantly different from Dermatology (H1) and Lab animal (H1). L12 for Surgeons in the Environment domain indicates that Surgeons is significantly different from Dermatology (H1), Lab animal (H1), and Radiology (H2).

Residents in ECC program had the lowest adjusted mean scores in all QOL domains: Psychological Health (35.1), Physical Health (45.8), Social Relationships (47.5) and Environment (52.2). Residents in Internal Medicine, Anesthesia and Analgesia, and Surgeons programs also had lower mean QOL scores across several domains compared with other programs. In contrast, residents in Lab Animal, Radiology, and Dermatology programs had higher mean QOL scores across several domains.

## Discussion

This study, to the best of the authors’ knowledge, is the first to measure the QOL of veterinary residents. This information yields important insight into the mental health of veterinarians during years of intense training, extensive working hours, professional growth, and rigorous preparation for specialty board exams. Our results demonstrate that there are differences in QOL among veterinary residents in AVMA-RVSOs based on specialty. This suggests that certain AVMA-RVSOs have a greater impact on an individual’s mental health than others. When comparing the four QOL domains, Psychological Health had the largest variation between programs. Laboratory Animal residents had the highest mean score (52.3) and ECC the lowest (35.1). The Psychological Health domain focused on questions involving the individual’s enjoyment of life, ability to concentrate, if they feel their life is meaningful, acceptance of bodily appearance, satisfaction with self, and frequency of negative feelings (blue mood, despair, anxiety, and depression). The next domain with the largest variation in scores was the Social Relationships domain with Radiology residents reporting a mean score of 63.4 compared to ECC residents with a mean score of 47.6. The Social Relationships domain focused on personal relationships, satisfaction with sex life, and satisfaction with the level of support from friends.

Mean standardized stratified results from a WHOQOL-BREF instrument population norms study in Canada for similar demographics (57% female, ages 30–39) are: 82 for Physical Health, 73.5 for Psychological Health, 74 for Social Relationships, and 73 for Environment domains [23]. These values are higher than the mean scores for all groups reported in this study, suggesting that veterinary residents have a lower QOL when compared to a general population with similar demographics. Programs focusing on improving residents’ self-esteem and encouraging growth of personal relationships are suggested for all specialties, with an emphasis for the lower scoring specialties. For example, it was demonstrated that regular group exercise for medical students can improve QOL and levels of self-care behaviors are linked to QOL in medical students [24, 25].

Similarly, medical students and individuals in medical residencies have been shown to have a lower QOL than the general population [26]. Brazeau [27] measured the QOL of students starting medical school and reported that matriculating medical students had higher scores when compared to a matched control group. They suggested that the training process and environment of medical school contributes to the deterioration of mental health in developing physicians [27].

Kay [28] found components of the WHOQOL-BREF instrument had strong predictive effects on sustained sad or hopeless feelings and suicidal behavior amongst college students in several Asian countries. While higher suicide risk is reported for veterinarians [4], research has indicated there is a reluctance for professionals to determine their own suicide risk suggesting an inadequate awareness of one’s own mental health [29]. Our study focusing on QOL of individuals in AVMA-RVSOs may help raise awareness for residents to self-assess their own mental health and help identify at-risk individuals.

There were several limitations with this study. The first is that survey studies are dependent on an individual’s willingness to participate as well as the honesty of the answers provided. Our utilization of a validated instrument, the WHOQOL- BREF, may optimize the validity and reliability of QOL measurements. All 22 AVMA-RVSOs were contacted requesting their assistance in disseminating the survey to their residents. AVMA-RVSOs were asked to email the link twice (several weeks apart) to active residents. Ten AVMA-RVSOs did not distribute the survey, with six of these reporting no active residents. This could have influenced the residents’ responses and rate of response. Social media was used to reach additional residents, but an unknown percentage do not participate in social media or may have ignored the unsolicited correspondence. The demographics of each AVMA-RVSO were not shared with the investigator. Some AVMA-RVSOs may not be aware of the number of residents they have at any given time. For this reason, we were unable to compute the true response rate and to compare our demographic results with those of each AVMA-RVSO. It is possible that our study population was different from the population of each specialty. Participation through an email link or a social media page was not differentiated. This might be a potential source of selection bias based on the participation of residents through email or social media. Further studies are required to identify other factors that may influence a resident’s QOL including year of residency, residency setting (university or private practice), and the time of year (e.g., immediately before examinations). Finally, longitudinal data are required to better assess the temporal relationship between the selection of specialty and QOL (i.e., whether the selection affects the QOL or vice versa) and how the selection of specialty affects the future QOL in veterinary residents.

The results of this study demonstrate the need for private institutions and universities to recognize the mental health challenges of their residents and develop supporting programs to improve QOL. Veterinary students and veterinarians may find that assessing different QOL domains improves one’s ability to more effectively care for their own overall mental health and well-being.

## Supporting information

S1 File CHERRIES checklist for reporting results of internet E-surveys. (DOCX)

S1 Data (XLSX)
