# Supplementary material for: Quality of life of veterinary residents in AVMA-Recognized Veterinary Specialty Organizations using the WHOQOL-BREF instrument
Source: PLoS One. 2022 May 12;17(5):e0268343. doi: 10.1371/journal.pone.0268343 (PMC9098004; doi:10.1371/journal.pone.0268343)
Supplement: S1 File — (DOCX) [file pone.0268343.s001.docx]

Checklist for Reporting Results of Internet E-Surveys (CHERRIES)

Item Category Checklist Item Explanation

Design Cross-sectional survey Veterinary residents in AVMA-

Recognized Veterinary Specialty

Organizations^TM^

IRB

(Institutional

Review Board) IRB approval Study was determined to

Approval and be Exempt by PearlIRB

Informed

Consent process Informed consent Survey was completed through

an online data collection service

(SurveryMonkey). Informed consent was provided in

the cover page and participants had to click agree before proceeding to the survey. SurveyMonkey alerted participants of the length of time of the survey, that the data would be collected/stored on the website. Participants were given the researchers personal email address and name. The purpose of the study was discussed along with the goal of publishing the data within the next year.

Data Protection Surveys were anonymous in order to protect unauthorize access

Development and testing WHOQOL-BREF, a validated instrument, was

used

Recruitment process Open survey, participants were

and description of the sample emailed by their specialty

having access to the questionnaire organization, or found the survey on social media which published it through veterinary specialty online groups

Survey administration The survey was available through SurveyMonkey (a website); responses were automatically captured

Context Participants were sent the link through their personal email, the survey link was also available on Facebook. Some residents may not use social media so they may not see a link available on Facebook.

Mandatory The survey was voluntary and participants could stop at anytime.

Incentives No incentives were offered

Time/Date April 2021- June 2021

Randomization of items No

or questionnaires

Adaptive questioning The first question would end the survey for people that did not self-identity as current veterinary residents in an accredited USA program

Number of Items Per page 1-10 questions per page

Number of screens 7 screens

Completeness check Yes; Surveymonkey alerts participant of completion. However, participants were allowed to skip questions or to stop at any time. Most responses provided a non-response option (Prefer not to answer, Neither satisfied or unsatisfied)

Review step Respondents could go to

previous page to review their

answers

Response rates

Unique site visitor Did not provide participation rate

Participation rate 865/1022 84.6%

Completion rate 792/865 91.6%

Preventing multiple entries Cookies and IP address was not used to track individuals

Analysis

Were only completed questionnaires analyzed? No

Were questionnaires terminated early also analyzed? Yes, The WHOQOL-BREF

Instrument provided details on how to grade their survey (which involved incomplete surveys)

Questionnaires submitted with an atypical timestamp No

Statistical correction All scoring was done based on

the WHOQOL-BREF instrument
